# Postoperative Radiotherapy for the Treatment of Solitary Fibrous Tumor With Malignant Transformation of the Pelvic: A Rare Case Report With Literature Review

**DOI:** 10.1097/MD.0000000000002433

**Published:** 2016-01-15

**Authors:** Chao Gao, Yong Zhang, Ming Jing, Wei Qu, Jia Li, Xiang-Rong Zhao, Yong-Hua Yu

**Affiliations:** From the Radiation Oncology Ward 2, Shandong Cancer Hospital and Institute (CG, YZ, WQ, JL, X-RZ, Y-HY); School of Medical and Life Sciences, Shandong Academy of Medical Sciences, Jinan University (CG, X-RZ); and Department of Dermatology, JiNan Dermatosis Prevention and Control Hospital, Jinan, Shandong, China (MJ).

## Abstract

Solitary fibrous tumor of the pelvic is an uncommon neoplasm with nonspecific symptoms. Reports of malignant transformation are especially rare. We report a case of solitary fibrous tumor in pelvic. A unique feature of our case compared with previously reported is that this patient relapsed with malignant transformation and had significant response to radiotherapy. The patient was initially treated with surgery, followed by postoperative dimensional conformal intensity modulated radiation therapy (dynamic MLC VRIAN 23EX Linac, inversely optimized by the Eclipse system) to provide a radical cure for residual tumor.

In this case, there were no signs of recurrence after six and a half years of further follow-up, indicating that postoperation radiotherapy may be an effective treatment for SFT with malignant transformation in pelvic.

## INTRODUCTION

Solitary fibrous tumor (SFT) is generally a benign neoplasm, the reported incidence of malignant SFTs varies from 7% to 60%, and the pelvis is a rare localization of SFT.^1^ Although nearly all SFTs have low malignant potential, malignancy can occur, especially if they grow to a large size or in the case of repeated recurrence.^2,3^ There are 2 forms of malignant transformation; one is malignant or high-grade SFT, and the other is de novo occurrence of malignant SFT.^4^

To our knowledge, the report of malignant transformation after recurrence of the SFT in pelvic is extremely rare. The main treatment for SFT is surgery. Herein, we report an SFT patient with postoperative recurrent malignancy transformation in pelvic. He was treated by radical surgical resection and subsequently underwent intensity-modulated radiation therapy (IMRT). It may provide a useful reference for the treatment of similar cases.

## CASE REPORT

**FIGURE 1:**
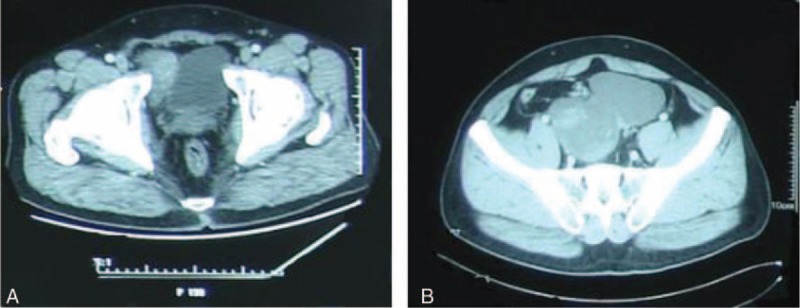
CT findings of the tumor in 2007. (A) CT showed a classical triangle soft tissue density shadow in the pelvic cavity; maximum cross-section was ∼5.5 × 2.5 cm. Lesions border was clear; its signal was well-distributed. Double side basin wall and inguinal region did not see obvious swelling lymph nodes; CT findings of the tumor at the second recurrence in 2009. (B) CT showed an irregular soft tissue mass shadow in the pelvic cavity. Lesions border was not clear. Local and right side of the bladder wall had not clear boundaries. CT = computed tomography.

**FIGURE 2:**
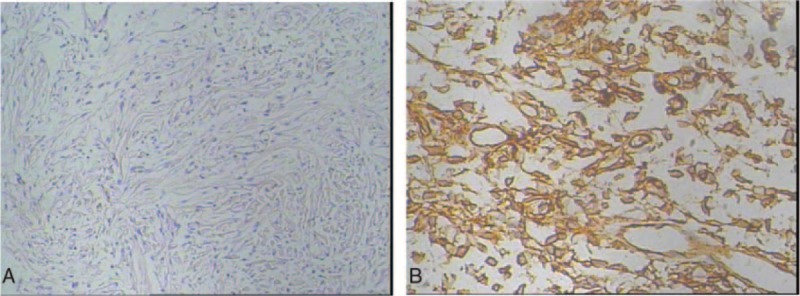
Histopathological findings of the primary tumor excised in 2007. (A) Spindle or oval cells with mild to moderate nuclear atypia, which shows a so-called patternless pattern (*a*: HE × 100). Immunohistochemical findings in 2007. (B) The specimen was positive for CD34.

A 58-year-old man was admitted to our hospital due to a pelvic mass, which was found by ultrasound in a private clinic in March 2007. Ultrasound demonstrated a large irregular marginated solid mass in the pelvic cavity. Computed tomography (CT) scan revealed a mass (5.5 cm in the longest diameter) in the cavity of the pelvic, the lesions border was clear, and no obvious swelling lymph nodes in double side basin wall and inguinal region (Figure 1A). Subsequently, the patient underwent surgical removal of the mass and the mass was removed completely. The surgery and postoperative period were uneventful. Pathologically, the tumor contained predominantly oval or spindle cells organized in a haphazard growth pattern (a so-called patternless pattern) (Figure 2A). Immunohistochemical stains revealed positive expression for CD34 (Figure 2B), Bcl-2, Ki-67, and Vimentin. Stains were negative for Desmin, S-100, SMA, and CD117. Finally, the tumor was diagnosed as SFT. Unfortunately, the patient refused any subsequent adjuvant treatment.

**FIGURE 3:**
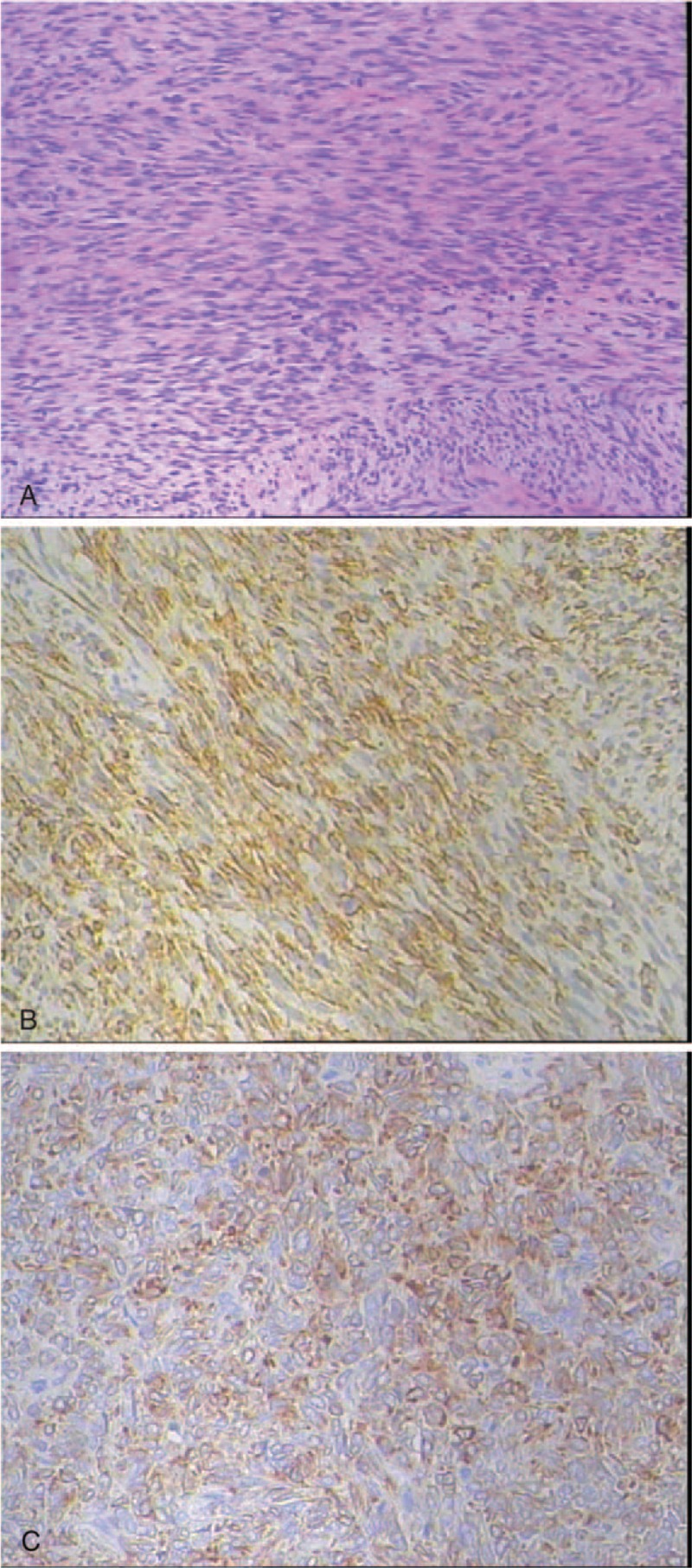
Histopathological findings of the tumor at the second recurrence in 2009. (A) Histologic evidence to support malignant transformation in solitary fibrous tumor of pelvic includes high cellularity with pleomorphism and increased mitosis, HE × 100. Immunohistochemical findings in 2009. (B) The specimen was positive for CD34. (C) The specimen was positive for Bcl-2.

He accidentally hit a mass in the right lower quadrant in February 2009. On the physical examination, the mass was hard, inactive, and painful if touched it. Laboratory tests, including tumor markers (CA-125, CEA, and CA-19-9), were within the normal range. Pelvic CT revealed a tumor of ∼7.5 cm in diameter, mixed density showing multiple soft tissue mass (Figure 1B). Subsequently, the patient underwent pelvic tumor resection again. During the surgery, the surgeons found the tumor, ∼13 cm in diameter, had severe adhesion to the lower abdomen abdominal and the surrounding tissue. Thus, a portion of the bladder was included in the resection. Resection proceeded and the postoperative course was uneventful. Postoperative pathology showed spindle cell tumor with hemorrhage and necrosis, and tumor cells had a certain degree of nuclear atypia, high mitotic activity (Figure 3A). Immunohistochemically, the specimen was positive for CD34 (Figure 3B), Bcl-2 (Figure 3C), CD99, and vimentin, but negative for S-100, SMA, and CD117. Combining with the medical history, it was considered as malignant SFT.

Due to the patient with recurrent malignant transformation, then we conducted post-operative IMRT to eliminate residual tumors, and avoid recurrence and/or metastases. Three weeks after the patient underwent operation, we developed a radiation treatment plan, and the patient underwent 3-dimensional conformal IMRT. The treatment machine was VRIAN 23 EX. LUNA TM 260 was used as the Eclipse planning system. The clinical tumor volume (CTV) was visualized on CT, and it was calculated including tumor bed. However, 0.3 cm outside of the CTV was defined as the planning target volume (PTV). We carried out the total PTV irradiation with a dose 60 Gy/30F, 2 Gy/F, 1 session/day, and 5 times/week. The DVH for each organ were as follows: bladder (D_max_ 65.8 Gy; D_mean_ 34.8 Gy), femoral head-R (D_max_ 49.4 Gy; D_mean_ 20.6 Gy), rectum (D_max_ 40.3 Gy; D_mean_ 22.0 Gy). After radiation therapy, no other obvious radiation reaction was observed except the slight cystitis. The patient had refused adjuvant chemotherapy and has been followed up for six and a half years. There was no clinical evidence of disease recurrence.

## DISCUSSION

SFT was first reported in 1931 by Klemperer and Rabin.^5^ It is a rare neoplasm that is thought to originate from submesothelial mesenchymal cells.^6^ The majority of SFTs are benign; however up to 20% of tumors have been reported to be malignant.^7^ Katsuno et al^8^ reported that 6% of all SFTs originated from the pelvis. Men and women appear to be affected equally and the tumor appears to develop primarily in middle age to older individuals.^9,10^ Systemic symptoms such as hypoglycemia, arthralgia, osteoarthritis, and clubbing have also been documented.^11,12^ In clinic, the symptoms of extrapleural lesions is usually associated with the location of tumor, and these symptoms could disappear when the tumor is removed.

Definitive diagnosis of SFT is based on the characteristic of histological and immunohistochemical features. Histologically, SFT is composed of spindle cells with oval nuclei, typically arranged in “patternless” arrangement, separated by thin “strip-like” bundless of collagen.^13^ The tumor may have noticeable but varying degrees of vascularity, and focal areas of blood vessels with a stag horn branching pattern characteristic of hemangiopericytomas are often found.^14^ Immunohistochemistry, the tumor cells exhibit expression for vimentin, CD34, CD99, and Bcl-2, but no reactivity for cytokeratin, S-100 protein, actin, desmin, and calretinin.^15,16^

According to the previous reports, CD34 is strongly positive for diagnosis of SFT,^17^ and Hasegawa et al^12^ suggested the both of Bcl-2 and CD34 immunostaining for making differential diagosis from spindle cell neoplasia. Although CD34 is a useful marker in diagnosis of SFT, 1 should bear in mind that its expression can be lost in high-grade tumours, in addition, overexpression of p53 and Ki-67 suggests the possibility of a malignant SFT, which relates to de novo or malignant transformation within a benign or low-grade.^4^ England et al^3^ considered neoplasms to be malignant if 1 or more of the following histologic features are present: (1) high cellularity, (2) high mitotic activity (>4 mitoses per 10 high-power fields), (3) pleomorphism, (4) necrosis, and (5) hemorrhagic changes. In the present study, the diagnosis of SFT was made according to the above characteristic immunohistochemical findings and histological features.

Currently, no standard therapy has been established for SFT with malignant transformation because of the rarity. The main method of treatment for SFT remains wide local excision of the tumor. Soft-tissue sarcomas have traditionally been managed by wide excisional surgery and radiation therapy, but in a study it was concluded that overall morbidity associated with adjuvant radiation was not significantly higher than that with surgery alone.^18^

The treatment for unresectable malignant SFT has not been established yet. Radiation therapy (RT) alone can be considered for patients refusing or unsuitable for surgery, and it can attain 30% to 60% rate of control in sarcoma.^19^ However, it is controversial if RT is therapeutic only for high-grade tumors or also for low-grade tumors.^20,21^ Kawamura et al^22^ reported a case of huge pelvic malignant SFT with lung metastasis, chemotherapy did not work whereas the tumor shrank 12 months after 50 Gy irradiation of the pelvic region. Moreover, in a series of 11 SFT treated with definitive RT without surgery, no patient had a local recurrence, and 9 were disease-free at 3 to 20 years from diagnosis.^23^ Furthermore, Saynak et al^13^ also reported RT alone achieved significant effect, which was consistent with Saynak M’s report.^13^ On the contrary, Dietrich et al^24^ indicated an accelerated progression of the solitary fibrous chest wall tumor in the course of irradiation. Whether the development of sarcomatic growth occurred as a result of RT remains speculative. Nevertheless, RT in SFT could be conceived when surgery is not suitable, as in meningeal, retroperitoneal, and pleural presentations, and pathologic signs of “malignancy” are present at the onset.

Postoperative adjuvant therapy with chemotherapy and/or RT has been used sporadically, but the benefit of these adjuvant therapies remains unproven. The literature is inconclusive in regard of adjuvant RT in SFT.^25–27^ Postoperative RT was given due to the high-grade malignancy, narrow excision margins, large size and rapid growing, but there are no published series to substantiate its routine usefulness for this disease.^28^ Xue et al^29^ reported four and a half years after postoperative RT, no local recurrences were observed. Furthermore, Wushou et al^30^ analyzed 227 SFT cases in the Surveillance, Epidemiology, and End Results database during the years of 2000 to 2009 and confirmed a survival benefit for patients treated with surgery in combination with adjuvant RT, whereas the effect was not appreciated with surgery alone. In another series, investigators found that the treatment protocol of combined adjuvant RT with surgery seemed to hinder tumor progression, but had no effect on overall survival.^31^

There are no standard chemotherapeutic indications or regimens for SFT. In some previous studies, Kawamura et al^22^ reported that SFT had no response to paclitaxel (4 sessions, 60 mg/m^2^), and the tumor had continued to increase 22.2% in size in the pelvic CT scan. Gold et al^2^ found that chemotherapy regimen of doxorubicin and cisplatin was also ineffective for the SFT patient. However, Grobmyer et al^32^ revealed that combination of doxorubicin, ifosfamide, and mesna was associated with a significant improvement in disease-specific survival in patients with high-grade extremity soft tissue sarcomas. Stacchiotti et al^33^ found that anthracyclines have a degree of antitumor activity in the range of soft tissue sarcoma chemotherapy, but ifosfamide monotherapy seemed to have lower activity. Furthermore, Park et al^34^ found that combination therapy with temozolomide and bevacizumab is a generally well tolerated and clinically beneficial regimen for SFT patients. On the contrary, Stacchiotti et al^35^ found that the temozolomede-bevacizumab combination did not induce a better therapeutic activity than temozolomide alone, and dacarbazine as a single agent has antitumor activity in SFT.

Several tyrosine kinase inhibitors have been reported, which have shown promise in treating SFT. For example, some previous studies reported that sorafenib has produced tumor responses in patients with SFTs.^36,37^ Stacchiotti et al^38^ found that sunitinib is also active in SFT, and response can be long-lasting. Additionally, malignant SFT can express platelet-derived growth factor receptor beta strongly and has missense mutation of 18 exons, providing promising treatment target for unresectable malignant SFT.^39^

In the present study, we conducted postoperative IMRT to eliminate residual tumors, which can better control the primary tumor and avoid recurrence and/or metastases, but the patient refused adjuvant chemotherapy again. No recurrence was observed in the subsequent follow-up, indicating that the patient could be benefited from postoperative RT and clinicians can be referenced from this strategy.

Our patient was initially diagnosed as benign SFT. After radical surgery, he refused to receive the subsequent adjuvant therapy with chemotherapy and/or RT, and did not go to the hospital regularly for re-examination. Currently, he had a relapse. The diagnosis was malignant SFT. Therefore, the first treatment of SFT was clearly insufficient. Following surgery, postoperative RT and chemotherapy should be performed in order to better control the primary tumor and avoid recurrence or metastases.

## CONCLUSION

In this case report, we described an SFT growing in the pelvic, an infrequent localization, which presented a unique associated with recurrent malignant transformation. The absence of chemotherapy and/or RT could be the reason of relapse. This patient underwent postoperation RT after recurrence, no recurrences or metastases occurred during long-term follow-up. Thus, postoperation RT may be an effective treatment for SFT with recurrent malignant transformation in pelvic. Additionally, chemotherapy also should be performed for the greatest degree to prevent relapse. Owing to the possibility of repeated recurrences or malignant transformation, a prolonged follow-up may be advisable in this rare disease.
